# Desirability and feasibility of value-based healthcare in the Dutch Military Health System: a cross-sectional study

**DOI:** 10.1186/s12913-026-14517-y

**Published:** 2026-04-11

**Authors:** Henk van der Wal, Fleur Maas, Iris Dijksma, Jan Hazelzet

**Affiliations:** 1 Trauma Research Unit, Department of Surgery, Erasmus MC, University Medical Center Rotterdam, Rotterdam, The Netherlands; 2 Defence Healthcare Organization, Ministry of Defence, P.O. Box 90004, Utrecht, 3509 AA The Netherlands; 3 Erasmus MC, University Medical Center Rotterdam, Rotterdam, The Netherlands; 4 Department of Public Health, Erasmus MC, University Medical Center Rotterdam, Rotterdam, The Netherlands

**Keywords:** Value-based healthcare, Military health system, Outcomes, Patient-centred care, Trauma care, VBHC, Large-scale combat operations, Familiarity, Feasibility, Desirability

## Abstract

**Background:**

Value-Based Healthcare (VBHC) emphasises delivering high-quality care measured by patient outcomes rather than the volume of services. Although mostly partially implemented in civilian health systems, VBHC integration within military health systems (MHS) lags behind. Given current geopolitical developments and the unique operational demands of military healthcare, this study aimed to assess the desirability and feasibility of implementing VBHC in the Dutch MHS.

**Methods:**

A cross-sectional, descriptive survey was conducted in 2024 among active-duty personnel in the Netherlands Armed Forces. Stratified sampling targeted three stakeholder groups—care recipients, care providers, and care facilitators—followed by voluntary response recruitment. A self-administered online questionnaire assessed familiarity with VBHC, perceived desirability and feasibility of its implementation, and prioritised components for application. The survey was structured around case-based scenarios derived from the Linnean VBHC framework, adapted to the military context. Quantitative data were analysed using descriptive statistics and Likert-scale medians; open-ended responses were analysed thematically.

**Results:**

Of 912 eligible participants, 290 completed the survey (32% response rate), comprised of 45% care recipients, 33% care providers, and 22% care facilitators. Most respondents (67%) had over 15 years of military service; 58% held higher education degrees. Familiarity with VBHC increased throughout the survey. Overall, 96% of respondents perceived VBHC as valuable for the Dutch MHS. Notably, 64% believed VBHC could benefit both regular and operational care. A collaborative implementation approach involving all three stakeholder groups was preferred by 82%. However, concerns were raised about feasibility in large-scale combat operations, highlighting the need to align VBHC components with military readiness and mission-critical objectives.

**Conclusion:**

This study provides initial evidence supporting broad acceptance of VBHC within the Dutch MHS, particularly for non-operational military healthcare contexts. While desirability was high, practical applicability raised concerns, especially regarding combat and operational care settings. These findings suggest the potential for VBHC integration, but emphasize the necessity for a military-specific adaptation. Future research should explore targeted implementation strategies that balance patient-centered outcomes with the unique operational demands of military healthcare environments.

**Supplementary Information:**

The online version contains supplementary material available at 10.1186/s12913-026-14517-y.

## Background

**Fig. 1:**
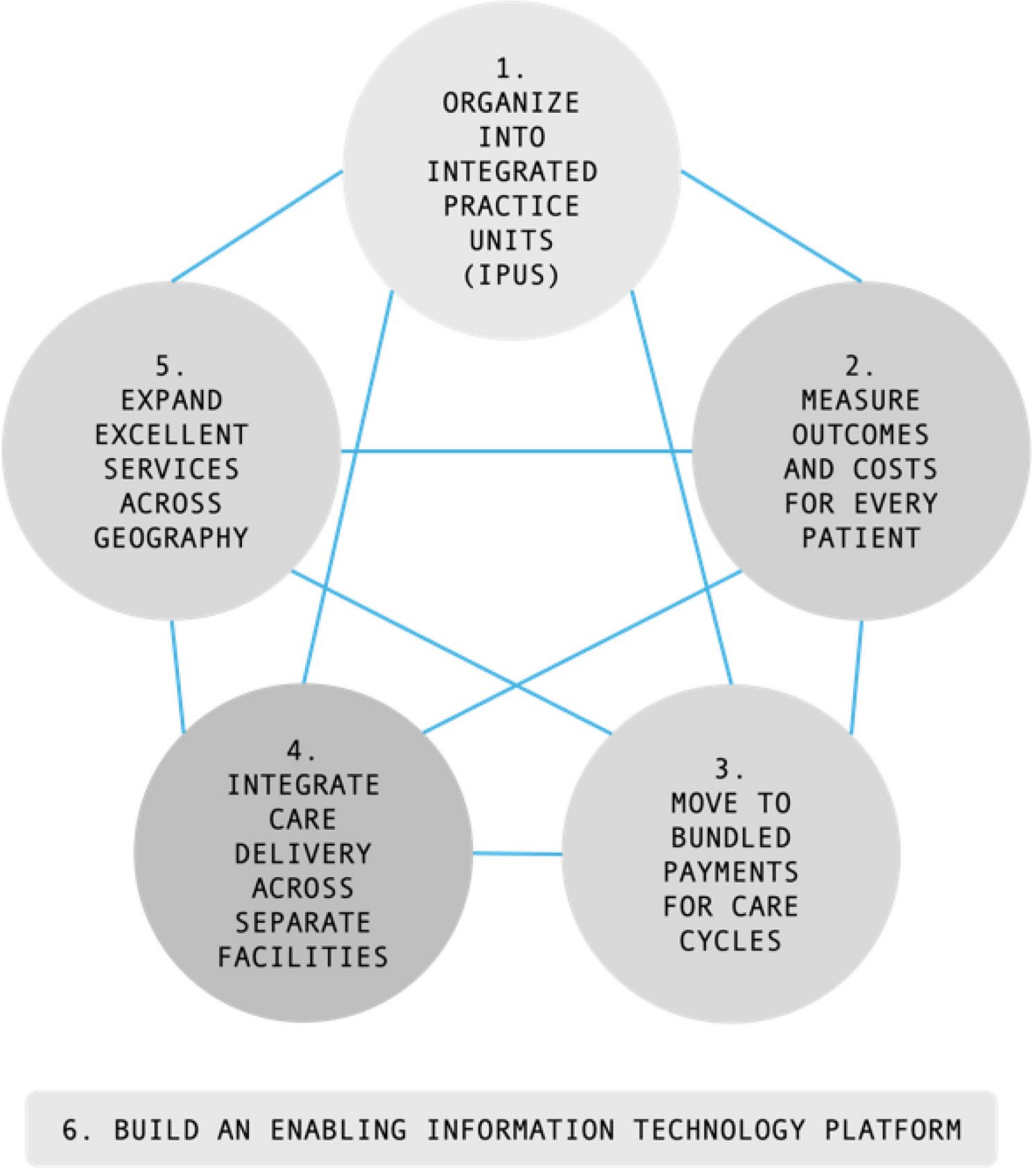
Porter’s value agenda components

**Value-based healthcare (VBHC)**, introduced by Porter and Teisberg in 2006, is focused on delivering high-quality care, measured by patient outcomes rather than the volume of services delivered [1, 2]. For years, a transformation to VBHC has been taking place on a larger scale in countries such as the United States of America, United Kingdom and also the Netherlands [3, 4]. In particular, a specific change strategy, shaped by the ‘Value Agenda’ [5], is implemented at local and institutional levels (e.g. hospital departments, and health networks). This strategic agenda for change towards high quality care delivery, consists of six interdependent and mutually reinforcing components (Fig. 1), with the greatest progress being made when more components are implemented simultaneously. However, the 2009 global financial crisis diverted health-system priorities towards immediate cost containment, slowing VBHC roll-out in many settings [6], and the COVID-19 pandemic further paused broad implementation as organizations pivoted to emergency responses and continuity of care [7].

**Fig. 2:**
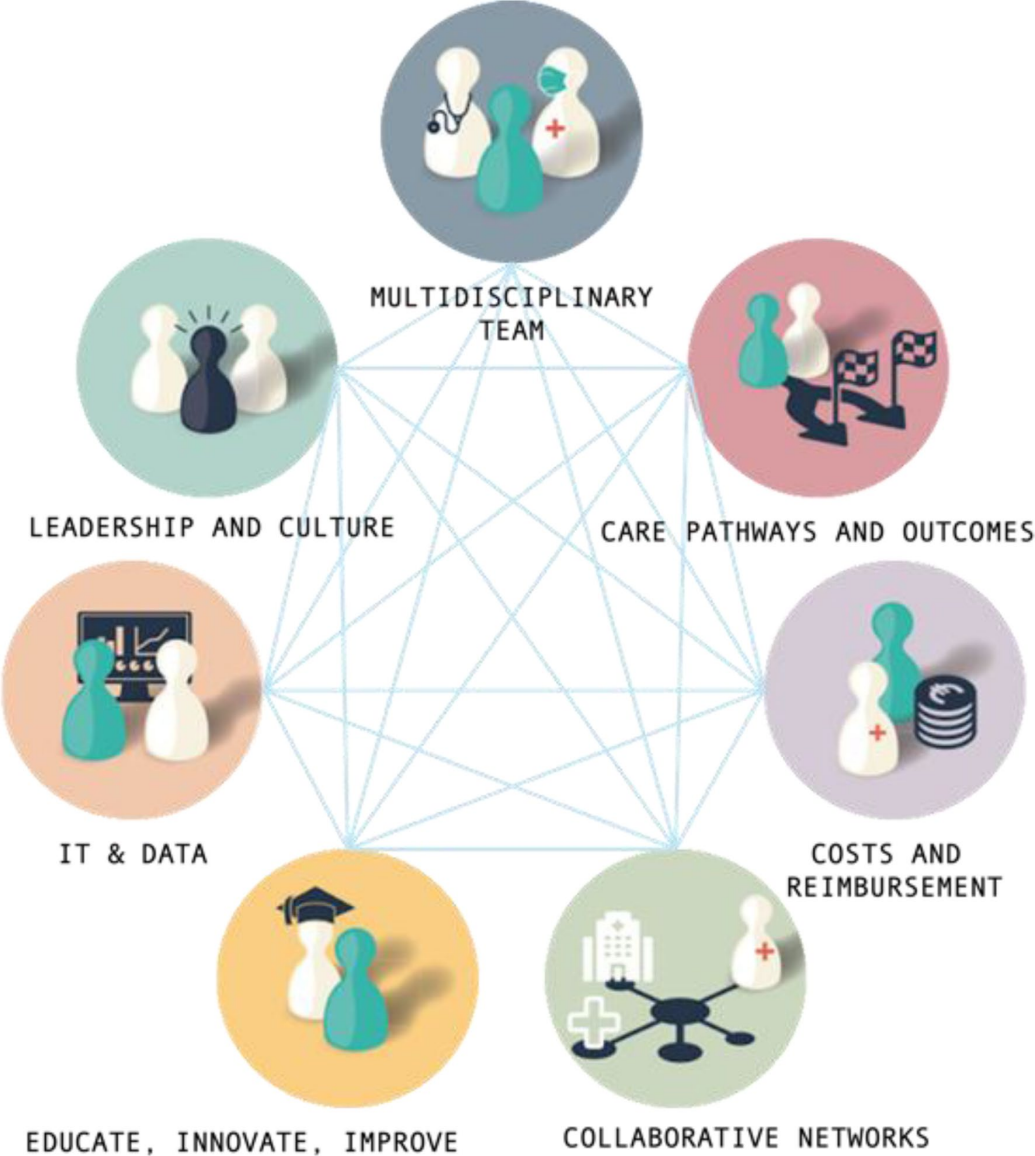
Linnean’s VBHC components

In the following years, the six interrelated components were further developed in the Netherlands by the collaborating network Linnean (8), towards: (1) ‘multidisciplinary team’ (2), ‘care pathways and outcomes’ (3), ‘cost and reimbursement’ (4), ‘collaborative networks’ (5), ‘training, innovation, improvement’ (6), ‘IT & data’, and (7) ‘leadership & culture’ (Fig. 2). Important additions to reinforce further operationalisation of VBHC are ‘patient-centred care’ and ‘shared decision-making’ [9, 10]. VBHC as a model is not yet integrated into military health systems, but implementation of individual components, as those mentioned above, is taking place [11]. Research has shown that in civilian healthcare, VBHC has a high level of interpretive variability with regard to how it is conceptualised, which means that with the diversity in civilian healthcare, implementation is also highly dependent on local choices [3]. At the hospital level, VBHC involves integrated, multidisciplinary teams accountable for a defined patient group, the transparent measurement of outcomes that matter to patients and the costs of delivering care in order to drive continuous value improvement. These data are used with patients and families in shared decision making. To optimise the use of VBHC and create added value by distinguishing it from other concepts, there is an urgent need for a common conceptualisation [3].

### Military healthcare

Military healthcare is available under all circumstances. This applies to the care that is standard in the Netherlands and at military locations, but also during military operations high on the violence spectrum and/or extremely difficult (climatic) conditions. This means that the military health system should also be seen as a model that addresses all aspects of health care interconnected and in a continuum. It should also connect and align with national and international stakeholders in (military) healthcare. The military healthcare system follows the North Atlantic Treaty Organisation’s (NATO) “Continuum of Care” model, prioritizing the fitness of service members after injury or illness [12, 13]. The focus is on preserving life, limb, and function while preparing for medical evacuation if necessary. Although the continuum is described as a patient-centred cycle, the system is more volume-driven than outcome-oriented. Geopolitical developments, particularly Russia’s invasion of Ukraine [14], have increased the need to enhance military healthcare capacity [15, 16]. Strengthening civil-military cooperation within the European Union (EU) and at national levels is crucial for cost-effective and sustainable defence [17].

Advancing VBHC particularly by improving the measurement of health outcomes at both the individual and (regarding the current geopolitical developments) population levels can support military medical leadership in enhancing the efficiency and effectiveness of the MHS aiming at maximising survival and minimising preventable mortality among injured service members [18]. Some VBHC pilots have also already taken place in the United States Military Health System, but these were mainly aimed at military regular care and not part of any transformation. However, the pilots did show a positive trend in quality, as well as a contribution to military readiness [19]. A May 2025 scoping review reported that most VBHC initiatives worldwide remain small-scale and called for stronger evidence on effective implementation models across different health-systems and resource settings [20].

The aim of this study is twofold: firstly, to assess the perceptions of VBHC within the Dutch MHS, and secondly, to explore the practical implications for implementation. The following research questions are addressed: (1) How is ‘VBHC’ currently perceived and understood within the Dutch military healthcare by military patients (care recipients), military healthcare professionals (care providers) and military healthcare leadership (care facilitators)?, and (2) What are the perceived levels of desirability and feasibility for implementing VBHC within the Dutch MHS?

## Methods

### Setting

The Dutch Military Health System (MHS) consists of two main care components: integrated care and operational care [21]. These elements ensure that service members receive appropriate medical support both on military locations in the Netherlands and during military operations. Integrated care is designed to maintain service members’ health and readiness. Each service member is assigned to a military medical team that provides general and occupational healthcare. Medical records are stored in an integrated system, accessible both in the Netherlands and during operations. Service members are required to use military healthcare services, but if specialized treatment is needed, they may be referred to civilian providers. In emergencies where military healthcare is unavailable, personnel can seek immediate civilian care. Operational care focuses on medical support in high-risk environments, such as deployments, training exercises, and conflict zones. It follows a medical evacuation chain, ensuring that injured service members receive treatment as they progress through different levels of medical support. Given the challenges of operational environments, military healthcare includes preventive measures, trauma care, and post-mission rehabilitation. Commanders conduct risk assessments before deployments, considering regional health threats and environmental hazards. Care may be provided by military medical units, civilian-military partnerships, or local healthcare facilities, depending on the situation.

In addition to what is mentioned in the aforementioned paragraph, it is important to recognise that current circumstances show that operational care is designed to provide the greatest chance of survival with the best possible outcome for the largest population [22]. To contextualize the following, it must be clarified what the main tasks of the Netherlands Armed Forces are: (1) defending national territory and that of our allies; (2) enforcing the national and international rule of law; and (3) providing assistance during disasters and crises [23]. In preparing for a ‘war of necessity’, the priority shifts from maximizing care for each individual—as seen over the past two decades during missions like those in Afghanistan and Iraq—to providing the maximum achievable care for the largest number of injured service members (i.e., doing the most for the most), which is currently the primary task.

### Study design

This cross-sectional, descriptive study was conducted in May and June of 2024. A stratified sampling method was used, followed by voluntary response sampling. The study targeted active-duty service members in the Netherlands Armed Forces. The reporting follows the Consensus-Based Checklist for Reporting of Survey Studies (CROSS) guidelines [24].

### Study population and sampling

Eligible participants included all active-duty service members across the Dutch Ministry of Defence. Stratification sampling was based on participant role: care recipients (service members as actual, past and potential patients, and users of the delivered services by the Dutch MHS), care providers (healthcare professionals, like physicians, nurses, allied health professionals, and physical therapists), and care facilitators (like leaders, managers, technical & logistical staff, and administrative staff). The sample size goal was over 1,000 respondents, accounting for a projected 35% response rate [25]. Only participants who fully completed the questionnaire were included in the analysis. Incomplete responses, as well as non-service members were excluded from the dataset.

### Survey development and instrument

A self-administered questionnaire was developed to assess perceived familiarity, desirability, and feasibility of VBHC within the Dutch MHS. The questionnaire was developed around the principles of two surveys: a perception survey [26], which focuses on subjective opinions and perceived advantages and disadvantages, and a stakeholder assessment survey [27], which gathers multi-perspective input for change based on participants’ roles. The survey was delivered using LimeSurvey (Dutch Defence version 1.91+, build 10746), and is available in both the original Dutch and translated English versions (see Supplementary Materials S1 & S2).

Part 1 of the questionnaire consisted of a brief introduction on VBHC and military healthcare. Followed by three demographic questions on the role, years of military service and education level of the respondent. This part ended with a 5-point Likert scale question on the level of familiarity with VBHC (scale: 1 (not at all), 2 (slightly), 3 (moderately), 4 (very), and 5 (extremely)).

Part 2 of the questionnaire presented seven case descriptions, each linked to specific components of the Linnean VBHC model, adapted to a military context [21, 28]. Each case included four to seven statements rated on a 5-point Likert scale for perceived importance (scale: 1 (not important), 2 (slightly important), 3 (moderately important), 4 (important), or 5 (very important)). Table 1 provides an overview of VBHC components and their associated case descriptions.

**Table 1:** Overview VBHC components used in case description

	Case descriptions						
	1	2	3	4	5	6	7
**Linnean component**							
Multidisciplinary team	X		X		X		
Care pathways and outcomes	X	X					X
Costs and reimbursements							X
Collaborative networks		X				X	
Educate, innovate & improve			X	X			
IT & data							X
Leadership & culture				X	X	X	

Part 3 of the questionnaire focused on the extent to which respondents had become familiar with VBHC. It then asked about the feasibility of implementing VBHC in the Dutch MHS. If the answer was ‘no’, respondents were asked why in an open-ended question. If they answered ‘yes’, they were asked in which part of the MHS (regular care, operational care, or both) VBHC could be applicable, how desirable the application of VBHC would be, how feasible the application of VBHC would be, and which components should be given the highest priority (open-ended question), The middle two questions using a 5-point Likert scale (1 (somewhat), 2 (slightly), 3 (desirable / applicable), 4 (very), and 5 (extremely). The use of the terms desirability (degree to which stakeholders perceive VBHC as an added value for the Dutch MHS) and feasibility (to what extent stakeholders believe in successful implementation) is based on Brown’s Principles of Design Thinking [29]. Finally, respondents were asked to select their preferred implementation strategy (bottom-up, top-down or collaborative).

The seven VBHC cases were developed by two of the researchers based on Linnean Initiative domain descriptions [8, 30, 31], adapted to the Dutch MHS context, and content-validated through expert review. The survey was pilot tested with five individuals (potential patients and researchers), and minor revisions were made for clarity based on verbal feedback.

### Data collection procedure and recruitment

Participants received as part of the overall questionnaire an informed consent form and brief introduction to VBHC before completing the questionnaire. Surveys were anonymous, and participation was voluntary.

### Recruitment

In May 2024, a three-subgroup sample of service members (explained under subheading study population and sampling) were asked via the Dutch Ministry of Defence (MoD) e-mail system to participate in this study by completing the included questionnaire. The list of potential participants was provided through the department within the MoD responsible for providing sample populations for various types of research. The provision of the e-mail was also the starting point of the data collection period. The e-mail contained a general introduction, the hyperlink to the questionnaire, instructions for participation and contact information of the principal investigator. A reminder e-mail was sent one week after the invitation e-mail. In order not to overload the participants with email traffic, it was decided not to send more reminder emails. One month after the invitation email, the data were collected from the online system and downloaded onto the principal investigator’s computer in a password-protected environment. The data collection period took place during May and June 2024.

### Analysis

Respondents’ demographics were tabulated as frequencies and percentages (n, %). For perceived knowledge, the median and interquartile range (IQR) of the ordinal ratings were calculated and distributions visualized using box-and-whisker plots. In part 2 of the questionnaire, the composite score of a component is obtained by summing the scores of all linked statements and dividing it by the number of linked statements, with higher scores indicating higher levels of importance. For the seven components of interest, median scores and IQR were calculated and displayed using Box-and-Whisker plots. Desirability and feasibility ratings were summarized using the median and IQR. All analyses were conducted using IBM SPSS Statistics V.30.0.0.0 (172). Categorical responses for applicability, preferred implementation strategy and grouped priority components were reported as frequencies and percentages. Open-ended responses were analysed using an inductive thematic analysis approach. First, all responses were read carefully to gain familiarity with the content. Key phrases and concepts were highlighted and grouped based on recurring ideas. These preliminary codes were then reviewed and consolidated into broader themes that captured the main barriers to implementing Value-Based Health Care (VBHC) within the Dutch Military Health System. The frequency of each theme was noted, and representative quotes were selected to illustrate the underlying sentiment of each theme. This qualitative coding approach enabled a structured yet flexible interpretation of the diverse responses [32].

## Results

**Fig. 3:**
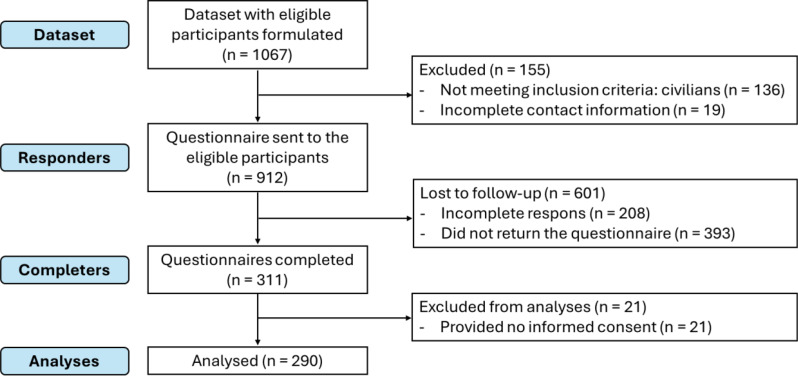
Participant inclusion flow-chart

A dataset with 1067 participants (consisting of 400 care recipients, 400 care providers, and 267 care facilitators) was set up, resulting in 912 questionnaires being sent after excluding civilians, and military personnel without actual military email address. After providing informed consent, 290 participants (response rate 32%) fully completed the questionnaire and were included in statistical analysis (Fig. 3). The demographic characteristics of the participants are summarized in Table 2.

**Table 2:** Demographic characteristics

	*N* (%)
Role within Defence	
- Care recipients - Care providers - Care facilitators	130 (45) 96 (33) 64 (22)
Years of experience within Defence	
- 0–5 years - 6–15 years - 15 + years	37 (13) 58 (20) 195 (67)
Level of education	
- Low education (ISCED 0–2) - Middle education (ISCED 3–4) - High education (ISCED 5–8)	15 (5) 107 (37) 168 (58)

**Fig. 4:**
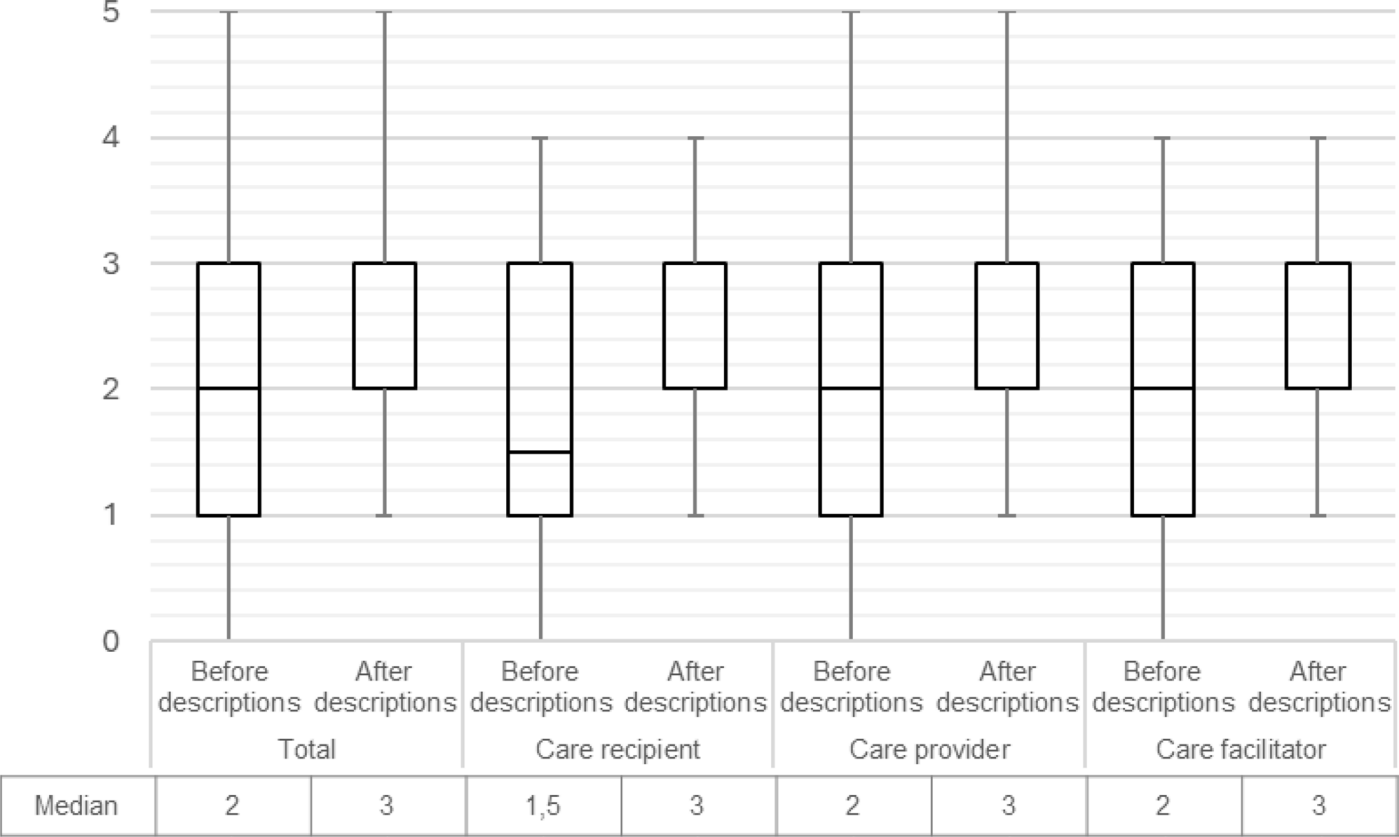
Box-Whisker plot on familiarity with VBHC before and after the provided case descriptions using median and interquartile range. Rating: 1 (not at all) – 5 (extremely)

**Fig. 5:**
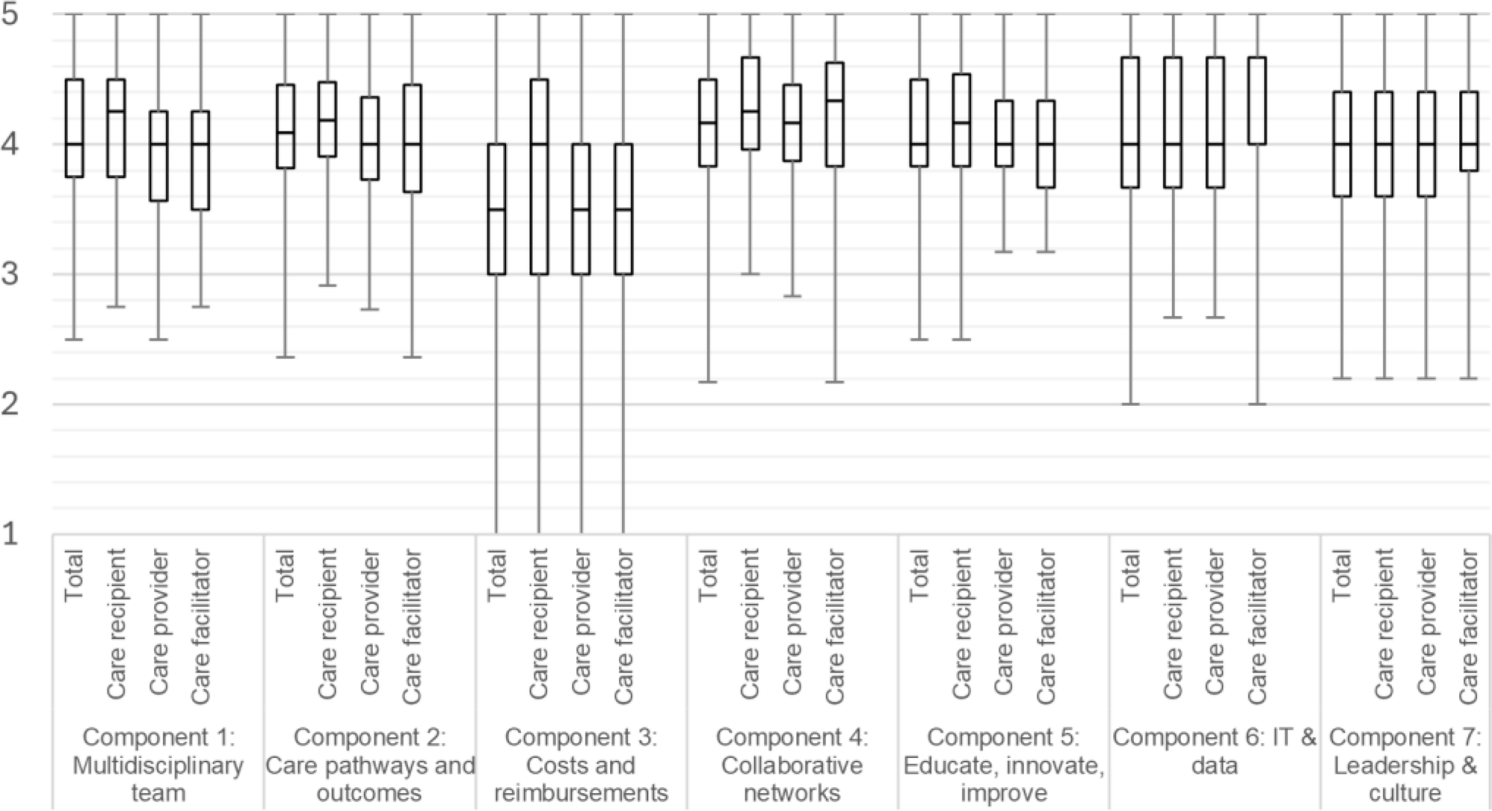
Box-Whisker plot using median scores with interquartile range to score importance for the seven components Rating: 1 (not important) – 5 (very important)

Figure 4 shows that there was an increase in knowledge of VBHC between the start and the end of the questionnaire. This trend is consistent across all three subgroups. The median scores for each component were mostly similar among the three groups and are presented in Fig. 5. Component 3, addressing costs and reimbursements, received consistently lower scores compared to the other components across all groups.

**Fig. 6:**
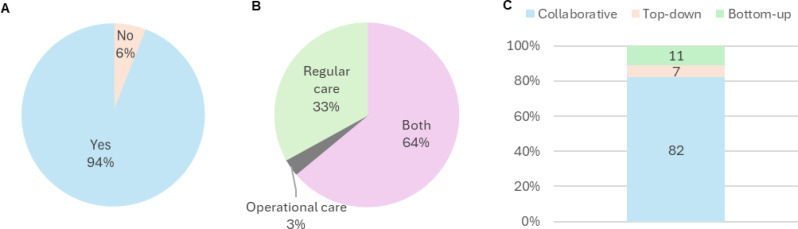
Participants’ perceptions of the value of VBHC within Dutch MHS: (**A**) overall perception of value, (**B**) perceived areas where VBHC may offer benefits; (**C**) implementation strategy VBHC model

Seventeen participants, five care recipients, six care facilitators, and six care providers, respectively, expressed that VBHC would not be of value within the Dutch MHS (Fig. 6A). Reasons given included concerns that VBHC may conflict with operational priorities, requiring significant adaptability and lacking clarity on “value” for military goals. Some questioned its relevance in large-scale combat operations and saw it as misaligned with military objectives, suggesting instead with a non-VBHC focus on perceived urgent issues like personnel costs and leadership stability (Supplementary material 3 (S3)). Illustrative comments included: “Not useful enough for operation care in a major conflict; principles are civilian” (respondent 5), “No need for everyone to know what I have; being sick can hurt your career–just get me back to duty” (respondent 8) and “Given the shift to main task 1, the pursuit of such a holistic approach is no longer possible…” (respondent 10). Among the remaining 273 participants, the majority indicated that the implementation of VBHC would be beneficial to both the regular and operational aspects of Dutch military healthcare, 33% perceived a benefit only for regular care, while 3% recognized an advantage only for operational care (Fig. 6B).

The median score ± IQR for desirability was calculated as 3 (desirable) ± 1, whereas the applicability received a score of 3 (applicable) ± 2. Elements that should be prioritized when implementing VBHC within Dutch MHS include aligning with military operational readiness, ensuring patient-centred care supports military goals, fostering multidisciplinary collaboration, enhancing data transparency and IT security, strengthening leadership, protecting patient privacy, and adapting VBHC to meet the unique demands of military healthcare settings (Supplementary material 4 (S4)).

Regarding the preferred implementation strategy for the model, 82% of respondents advocated for a collaborative approach, while 7% supported a top-down strategy, and 11% favoured a bottom-up strategy (Fig. 6C).

## Discussion

This study aimed to explore the desirability and feasibility with VBHC amongst military care recipients (patients), care providers and care facilitators in the Dutch MHS. The results suggest that despite low familiarity with VBHC, even a brief exposure to the concept may trigger a strong interest in the applicability of (parts of) VBHC in the Dutch MHS. Also, the recurrent question on familiarity with VBHC showed an increase from initially slightly familiar to almost moderately familiar. After introducing the VBHC concept using the seven case descriptions, participants saw VBHC as desirable and feasible in both military operational and military regular care. About two-thirds of that group of participants saw an application in military healthcare as a whole. In addition, about one-third considered VBHC applicable only in regular care and some participants only applicable in operational care. When the results to the aforementioned were distributed to the subgroups of care recipients, care providers and care facilitators, it was found that despite the variation in demographics, the subgroups scored largely the same on perceived importance in descriptions, as well as on desirability and feasibility. This level of desirability and feasibility demonstrates that a focused and phased approach could enable integration of (parts of) VBHC into an MHS.

These results indicate a clear difference in how participants perceive desirability and applicability, with desirability receiving higher ratings than applicability. This indicates that, while participants may find the concept desirable, they may see challenges or barriers in its applicability in practice. This gap between desirability and applicability could be an area for further investigation, possibly focusing on identifying and addressing specific barriers to applicability. In addition, participants expressed concerns that VBHC may conflict with operational priorities, requiring significant adaptability and lacking clarity on “value” for military goals. Some questioned its relevance in large-scale combat operations and saw it as misaligned with military objectives, suggesting instead a focus on urgent issues like personnel costs and leadership stability. Research shows that with the preparation of large-scale combat operations, the quality of military medicine should not be out of the scope but more explicit attention should be paid to issues such as delayed evacuations, logistical resupply, simpler and faster triage, all around massive numbers of combat casualties [33].

The above-mentioned barriers and concerns also show that service members want to participate and have an opinion on issues that affect the military healthcare as a whole. This was evident in the survey results where respondents expressed a desire to participate in thinking about possible developments for their MHS. This can also be interpreted as involvement (military involvement in the Netherlands Armed Forces in Q2 2024 68% [34]) and loyalty to their own immediate environment and to the armed forces [35]. Within the possibilities of the questionnaire, besides barriers, possible solutions to those barriers were also mentioned. This shows itself in the named barrier that Defence focuses on the readiness and deployability of military personnel, which can lead to situations where the focus is no longer on the patient. This manifests itself in the aforementioned ‘doing the most for the most’. However, if VBHC is nevertheless implemented in the Dutch MHS, a new component should be added. This component should focus on the military operational context, where in addition to focusing on the individual patient, deployment readiness should also be addressed.

Whereas a certain degree of solution orientation including suggestions could already be observed with the barriers, this is also reflected in the question on prioritising elements discussed in the case descriptions. This is further substantiated by the increase in familiarity with VBHC as surveyed at the end of the questionnaire. Of the elements that have been given high priority, a key observation is patient centeredness. This was recognised by respondents based on the knowledge provided in the questionnaire. However, it appears that patient-centred care is only supported by VBHC to a limited extent [36]. Furthermore, the application of VBHC was again discussed in relation to the main tasks of the Netherlands Armed Forces. The respondents see no or only limited possibilities for the application of VBHC during operations under main task 1. Any application in main task 1 must be accompanied by explicit military operational considerations.

From an overall perspective, apart from the VBHC component ‘Costs and reimbursement’, all other components were addressed, focusing mainly on the application in military regular care and, if possible, also in military operational care. Across the three groups of respondents, issues such as IT, data and dashboards (related to ‘IT & Data’) were highlighted, as well as the need for complete military care pathways (related to ‘Care Pathways and Outcomes’) and (inter)national civilian and military collaboration (‘Collaborative Networks’). By contrast, other studies show that there is interest in implementing VBHC components [37, 38], but this is typically not done using an integrative strategy [3]. One quote about the use of VBHC in the Dutch MHS should not go unmentioned, as it outlines the committed contribution of the respondents: “It’s worth the challenge”.

The above demands considerations from a warfare context. Lessons can be learned from the circumstances in Ukraine, such as despite being at war applying a change management approach in order to better align with the situation. One of the chosen mechanisms is therefore expanding the delivery of services through external (civil) partnerships, both (inter)national and with NGOs and healthcare institutions [39]. The planned NATO Research Task Group on Military Value‑Based Health Care (VBHC), expected to begin in autumn 2025, creates a multinational forum to extend our findings into coordinated implementation. Because respondents endorsed VBHC most strongly for routine (non‑operational) military care and only conditionally for operational contexts, follow‑up NATO‑level research should: (1) co‑develop and validate a core military VBHC outcome set with common data standards that interoperate with national civilian systems; (2) conduct multi-country pilot studies comparing VBHC‑aligned and usual‑care pathways across routine care, deployment preparation, and selected operational/mission‑exercise settings, tracking transitions between military and civilian care; and (3) evaluate secure, scalable information solutions that link patient‑reported and clinical outcomes to readiness, recovery, and resource use for alliance‑wide benchmarking and learning. Progress along these lines would help translate VBHC principles into improved force health protection under current geopolitical demands.

In light of the persistent challenges confronting health systems on a global scale, there is an imperative for the development of innovative frameworks such as VBHC, which have the potential to enhance patient outcomes [20]. It is imperative that the findings of this study should be utilised beyond the confines of the military-specific paradigm.

### Limitations

 A limitation of this study was that the overall response rate was only 32%, leaving two-thirds of invited service members without a completed survey. About 50% opened the questionnaire and roughly 20% began but did not finish, often citing its length, perceived difficulty, or lack of relevance. Response rates also differed by subgroup—care recipients 45%, care providers 33%, and care facilitators 22%—resulting in uneven cell sizes and limiting the robustness of subgroup comparisons. Although the instrument was designed to capture multi-perspective perceptions, these differential response rates limit the robustness of subgroup comparisons. That said, a 32% participation level compares favorably with US Department of Defense active-duty surveys, which have seen response rates decline from approximately 40% in 2004 to about 15% by 2018 [40]. Thus, while differential participation remains a consideration, our response rate aligns with—and in many cases exceeds—comparable military surveys.

Incomplete survey records were missing > 5 items and often entire VBHC components; imputing such extensive missingness would have produced model-driven, potentially biased component computed scores, so we analysed complete cases and acknowledge possible nonresponse bias. The achieved respondents sample included a high proportion of highly educated respondents, which may reflect the selective participation by individuals more engaged with VBHC. Representativeness is therefore uncertain, and findings should be interpreted as exploratory.

Despite the fact that this study is about the perceived desirability and feasibility of VBHC in the overall Dutch MHS, the responses of the care recipients and care providers are most important [41, 42]. Furthermore, the limited knowledge of the respondents and the limited application of VBHC in the Dutch military healthcare at present must be taken into account, which may limit the interpretation of VBHC to enable application. Respondents also had limited time to acquire knowledge about VBHC. Subsequently, this study used one survey moment, so only a baseline measurement took place, and no further trends can be predicted. Finally, since this was a first exploration and would therefore give only a baseline indication, further validation of the questionnaire should be performed.

## Conclusions

In a survey of active-duty Dutch military personnel spanning care recipients, providers, and facilitators, we found broad support for implementing VBHC in the Dutch MHS. Support was strongest for adoption on regular (non-operational) military care aimed at improving health outcomes and force readiness. Respondents also saw potential for applying elements of VBHC in operational care, but flagged concerns about feasibility under demanding operational conditions and the Armed Forces’ primary mission tasks. To our knowledge, this is the first study to bring together perspectives across these rolls, providing an empirical baseline for targeted implementation research, including work on linking health outcomes to individual readiness and recovery.

## Supplementary Information

Below is the link to the electronic supplementary material.

Supplementary Material 1

Supplementary Material 2

Supplementary Material 3

Supplementary Material 4

## Data Availability

All data generated or analysed during this study are included in this published article, and its supplementary information files. Data sharing is not applicable, as no datasets were generated and/or analysed for this study. All data relevant to the study are included in the article or uploaded as online supplemental information.

## Abbreviations

CROSS: Checklist of Reporting of Survey Studies
EU: European Union
IBM: International Business Machines Corporation
IQR: Interquartile ranges
ISCED: International Standard Classification of Education
IT: Information Technology
MHS: Military Health System
NATO: North-Atlantic Treaty Organization
NGO: Non-governmental Organisation
SPSS: Statistical Package for the Social Sciences
VBHC: Value-Based Healthcare
