# Supplementary material for: Desirability and feasibility of value-based healthcare in the Dutch Military Health System: a cross-sectional study
Source: BMC Health Serv Res. 2026 Apr 11;26:735. doi: 10.1186/s12913-026-14517-y (PMC13196245; doi:10.1186/s12913-026-14517-y)
Supplement: Supplementary file 1 — Supplementary Material 1 [file 12913_2026_14517_MOESM1_ESM.pdf]

Supplemental material 1 (S1) - Original questionnaire in Dutch language

DEEL 1 – INTRODUCTIE

Korte introductie over Waardegedreven zorg (WGZ) en de Militaire Gezondheidszorg (MGZ)

Het concept 'Waardegedreven Zorg' (in het Engels: Value-Based Healthcare) is in 2006 geïntroduceerd door prof. Michael Porter en prof. Elisabeth Teisberg. Samengevat komt waardegedreven zorg hierop neer: 'patiënten de best mogelijke zorg bieden en die zij zelf belangrijk vinden in een betere verhouding tot de werkelijk gemaakte kosten'. Binnen de Nederlandse gezondheidszorg krijgt WGZ een steeds belangrijkere plaats. Patiënten worden actiever betrokken bij hun zorgproces en daarmee ook bespreekbaar maken van hun specifieke gezondheidsdoelen met hun zorgverlener. Onderzoek wijst op positieve effecten voor zowel patiënten als zorgverleners, en het draagt bij aan duurzaamheid in de gezondheidszorg.

In contrast hiermee is de MGZ met name gericht op het gereedstellen en handhaven van de gevechtskracht door het waarborgen van geneeskundige zorgcapaciteiten voor militairen. De enquête onderzoekt de haalbaarheid van waardegedreven zorg binnen de MGZ, waarbij het WGZ-concept zich o.a. onderscheidt door de patiënt deelnemer te maken van het multidisciplinaire (zorg)team en de focus te leggen op persoonlijke zorgbehoeften.

Het multidisciplinaire WGZ-team bestaat uit een direct zorgteam (artsen, verpleegkundigen, tandartsen, fysiotherapeuten, praktijkondersteuners, etc.), een indirect zorgteam (administratief personeel, managers, leiders, technisch & logistiek personeel, etc.) én patiënten. Patiënten zijn immers dé experts van hun eigen leven en weten als geen ander wat waardevolle zorg voor hen inhoudt. Afhankelijk van de medische conditie kunnen ook de naasten van de patiënt, zoals partner, ouders en kinderen, en/of de mantelzorger(s) betrokken worden. De scheiding van het directe en indirecte zorgteam waarborgt de privacy en het medisch beroepsgeheim. De betrokkenheid van de patiënt wordt actief aangemoedigd.

Allereerst vier vragen over uw achtergrond binnen defensie en bekendheid met WGZ. Kruis aan wat het meest van toepassing is.

1. Waar bent u werkzaam binnen Defensie?
    - Indien niet werkzaam binnen de Militaire Gezondheidszorg: Militaire patiënt (een zorgontvanger)
    - Indien werkzaam binnen de Militaire Gezondheidszorg: Werkzaam als zorgverlener in een direct zorgteam (een zorgverlener)
    - Indien werkzaam binnen de Militaire Gezondheidszorg: Werkzaam als zorgondersteuner in een indirect zorgteam (een zorgondersteuner)

**Indien u niet werkzaam bent binnen de de MGZ, dan verzoeken wij u te kiezen voor keuze 'Militaire patiënt', om zodoende de vragen te beantwoorden vanuit uw rol als (potentiële) zorgontvanger.*

2. Hoeveel ervaring heeft u in totaal binnen defensie?
    - 0 - 5 jaar
    - 5 - 15 jaar
    - 15+ jaar

3. Wat is uw hoogst behaalde diploma?
    - Geen diploma
    - Basisonderwijs
    - VMBO, HAVO / VWO onderbouw, MBO1
    - HAVO, VWO, MBO2-4
    - Bachelor (HBO / WO)
    - Master (HBO / WO)
    - Doctor, PhD

4. Op een schaal van 1 (helemaal niet bekend) tot 5 (buitengewoon bekend), in hoeverre bent u al bekend met waardegedreven zorg?
   Schaal: 1-2-3-4-5

**1 - Helemaal niet bekend**	**2 - enigszins bekend**	**3 - redelijk bekend**	**4 - zeer bekend**	**5 - buitengewoon bekend**

DEEL 2 - BESCHRIJVINGEN VAN WAARDEGEDREVEN ZORG

Zeven beschrijvingen over WGZ in een militaire context

Nu volgen zeven beschrijvingen van een of meer deelaspecten van WGZ, toegepast binnen de MGZ. U wordt op basis van de beschrijvingen gevraagd in hoeverre u de genoemde uitspraken belangrijk vindt. Bij vraag één heeft u een rol gekozen (zorgontvanger, zorgverlener of zorgondersteuner). Het verzoek is om de rest van de vragenlijst op basis van uw mening, ervaring en gekozen rol in te vullen.

Beschrijving 1. De militaire gezondheidszorg heeft als doel de gereedheid van de militair te verbeteren. Dit gebeurt binnen het militair geneeskundige zorgpad waarin de zorgactiviteiten worden beschreven die kunnen plaatsvinden in Nederland (reguliere zorg) en tijdens missies en oefeningen (operationele zorg). Waar mogelijk zullen de patiënt en de zorgverlener gezamenlijk beslissingen nemen over diagnostiek en behandeling, gebaseerd op medische noodzaak, beschikbaarheid van middelen en mogelijkheden, en behoefte van de patiënt.

Om de beste gezondheidsuitkomsten te bereiken worden patiënt-gerapporteerde uitkomsten (zoals pijn en mentaal welzijn) en klinische gegevens (zoals bloeddruk en temperatuur) door de zorgverlener en patiënt gebruikt bij het maken van deze keuzes. Het krijgen van de beste gezondheidsuitkomsten vereist multidisciplinaire zorg vanuit een team met artsen en verpleegkundigen, maar ook ondersteunend personeel en commandanten. En zeker niet te vergeten de patiënt en zijn of haar partner.

5. **Geef voor de volgende 5 uitspraken aan hoe belangrijk u de aandacht voor het gestelde vindt binnen de MGZ?**
   (Schaal: ZB-zeer belangrijk / B-belangrijk / RB-redelijk belangrijk / EB-enigszins belangrijk / O-onbelangrijk)

Nr.	Uitspraak	ZB	B	RB	EB	O
1.	Patiënten (én partner) en zorgverleners beslissen samen (shared decision making)					

2.	Het multidisciplinaire team heeft gezamenlijke verantwoordelijkheid voor de gezondheidsuitkomsten					
3.	Patiënt staat centraal					
4.	Patiënt-gerapporteerde uitkomsten dragen bij aan betere gezondheidsuitkomsten					
5.	Het zorgpad is bekend en zichtbaar in een dashboard.					

Beschrijving 2. Dashboards geven in één oogopslag inzicht in de medische activiteiten in het militaire zorgpad en de door de militaire patiënt gerapporteerde uitkomsten, zowel in Nederland als tijdens missies en oefeningen. Het dashboard helpt de patiënt en de zorgverlener om de keuzes voor diagnostiek en behandeling aan te sluiten bij de behoefte van de patiënt. Ook kan een dashboard helpen bij het verbeteren van de kwaliteit van zorg en efficiënt inzetten van, de vaak schaarse, zorgmiddelen. Dit is allemaal gericht op het leveren van de best afgestemde zorg met de beste gezondheidsuitkomst voor de militaire patiënt. Het is belangrijk dat iedereen in het militaire zorgpad, vooral de patiënt en zijn of haar partner, weet wie de hoofdbehandelaar is, wie de contactpersoon is en waar 24/7 hulp beschikbaar is.

6. **Geef voor de volgende 6 uitspraken aan hoe belangrijk u de aandacht voor het gestelde vindt binnen de MGZ?**
(Schaal: ZB-zeer belangrijk / B-belangrijk / RB-redelijk belangrijk / EB-enigszins belangrijk / O-onbelangrijk)

Nr.	Uitspraak	ZB	B	RB	EB	O
1.	Dashboards zijn ook beschikbaar voor de patiënt					
2.	Het multidisciplinaire team werkt samen met iedereen in het militaire zorgpad van de patiënt					
3.	Het militaire zorgpad gaat over het totaalpakket aan zorg voor de patiënt					
4.	Het is voor de patiënt (én partner) altijd duidelijk wie de hoofdbehandelaar is					
5.	Het is voor de patiënt (én partner) altijd duidelijk waar en 24/7 voor hulp aan te kloppen					
6.	Uitkomsten moeten duidelijk en simpel worden weergegeven in dashboards					

Beschrijving 3. De zorgverleners in de militaire gezondheidszorg willen elke dag de kwaliteit van de zorg verbeteren en zichzelf ontwikkelen. Zij vinden het belangrijk om te blijven leren, niet alleen van de patiënten, maar ook van elkaar en van de (inter)nationale civiel-militaire gezondheidszorg. Het multidisciplinaire team deelt gezondheidsuitkomsten met andere zorgverleners, commandanten en instanties in het zorgpad. Dit delen is bruikbaar om de zorg verder te optimaliseren en/of vernieuwen

en altijd gericht op betere gezondheidsuitkomsten en kwaliteit van leven voor de patiënt. De leider (of het leiderschapsteam) is verantwoordelijk voor dit verbeterproces en het optimaliseren van deze uitkomsten en dashboards.

7. **Geef voor de volgende 5 uitspraken aan hoe belangrijk u de aandacht voor het gestelde vindt binnen de MGZ?**
(Schaal: ZB-zeer belangrijk / B-belangrijk / RB-redelijk belangrijk / EB-enigszins belangrijk / O-onbelangrijk)

Nr.	Uitspraak	ZB	B	RB	EB	O
1.	Zorgverleners verbeteren zich in hun vak					
2.	Het multidisciplinaire team gebruikt gezondheidsuitkomsten om te leren en te verbeteren					
3.	Onderzoeksresultaten zijn ook beschikbaar voor de patiënt					
4.	Patiënt (en partner-) ervaringen zijn onderdeel van het leer- en innovatieproces					
5.	Het multidisciplinaire team is ook verantwoordelijk voor effectief gebruik van middelen					

Beschrijving 4. Defensie verplicht militairen om de militaire gezondheidszorg te gebruiken. Deze wordt geleverd door de militair geneeskundige dienst of, na doorverwijzing, door de civiele gezondheidszorg. De beste zorg wordt geleverd wanneer het multidisciplinaire team samen de verantwoordelijkheid neemt voor het gehele militaire zorgpad. Hierbij zijn samenwerking, vertrouwen, duidelijkheid en het leren van fouten belangrijk. Het leiderschap toont moed en ambitie om de zorg te verbeteren en zorgt voor een cultuur van altijd leren en beter worden.

8. **Geef voor de volgende 5 uitspraken aan hoe belangrijk u de aandacht voor het gestelde vindt binnen de MGZ?**
(Schaal: ZB-zeer belangrijk / B-belangrijk / RB-redelijk belangrijk / EB-enigszins belangrijk / O-onbelangrijk)

Nr.	Uitspraak	ZB	B	RB	EB	O
1.	Vertrouwen, leren en innovatie zijn belangrijk binnen het zorgteam.					
2.	Onderzoek is gericht op betere zorg voor een specifieke patiëntengroep.					
3.	Zorgverlening is een teamprestatie.					
4.	Onderzoeksresultaten zijn beschikbaar voor andere instellingen.					

5.	De leider is verantwoordelijk voor de sfeer binnen het zorgteam.					

Beschrijving 5. Het zorgteam is verantwoordelijk voor een goede cultuur rondom het militaire zorgpad. De leider moet ervoor zorgen dat iedereen samen verantwoordelijk is voor gezondheidsuitkomsten en kosten. Door betere uitkomsten, efficiëntere zorg en lagere kosten te behalen, kan er meer zorg beschikbaar komen. Het team moet ook militaire leiders en beleidsmakers buiten de militaire zorg betrekken in een cultuur die draait om het leveren van de beste zorg. Deze partijen kunnen dan faciliteiten, inclusief financiële steun, aanbieden om de zorg en de door de militair (én partner) gekozen gezondheidsuitkomsten te verbeteren.

9. **Geef voor de volgende 4 uitspraken aan hoe belangrijk u de aandacht voor het gestelde vindt binnen de MGZ?**
(Schaal: ZB-zeer belangrijk / B-belangrijk / RB-redelijk belangrijk / EB-enigszins belangrijk / O-onbelangrijk)

Nr.	Uitspraak	ZB	B	RB	EB	O
1.	Patiënten (en partner) zitten ook in het multidisciplinair team.					
2.	De leider heeft lef, ambitie en neemt verantwoordelijkheid.					
3.	Militaire leiders en beleidsmakers worden actief betrokken bij het zorgproces.					
4.	Het multidisciplinaire team heeft gezamenlijke verantwoordelijkheid voor de kosten.					

Beschrijving 6. Leiderschap en cultuur zijn belangrijke elementen voor de prestaties van het team. Het gewenste doel kan vanuit twee invalshoeken worden bekeken, zowel vanuit de organisatie als vanuit de militaire patiënt. Organisatorisch werkt het team als één aan de beste resultaten om binnen het militaire zorgpad de gewonde met de juiste zorg snel af te voeren. Vanuit de patiënt wordt gekeken of de uitkomsten écht goed zijn met behulp van patiënt-gerapporteerde uitkomsten, zoals een pijnschaal of het functioneren in het werk. Deze aanpak geldt voor het vergelijkingen met andere instellingen in de reguliere zorg, maar ook voor de samenwerking met internationale militaire partners tijdens militaire inzet. Zo'n aanpak helpt bij het bevorderen van een cultuur van veiligheid, leren en verbeteren.

10. **Geef voor de volgende 5 uitspraken aan hoe belangrijk u de aandacht voor het gestelde vindt binnen de MGZ?**
(Schaal: ZB-zeer belangrijk / B-belangrijk / RB-redelijk belangrijk / EB-enigszins belangrijk / O-onbelangrijk)

Nr.	Uitspraak	ZB	B	RB	EB	O
1.	Samenwerking tussen het zorgteam en netwerkpartners is belangrijk voor de uitwisseling tussen kennis en ervaringen.					

2.	Individuele teamleden hebben inzicht in het gehele zorgpad.					
3.	Keten- en netwerkpartners helpen om de zorg patiëntgericht en gestroomlijnd te laten verlopen.					
4.	Patiënt-gerapporteerde uitkomsten zijn belangrijk.					
5.	Er moet meer worden samengewerkt met externe en internationale (militaire) partners.					

Beschrijving 7. Het militaire zorgpad wordt ondersteund door data, zodat zorgverleners, patiënten en anderen goede medische en organisatorische beslissingen kunnen nemen. Dit gebeurt veilig via een IT-systeem. Een IT-platform zorgt voor betrouwbare informatie die op tijd beschikbaar is en zichtbaar is via dashboards. Zo kunnen beslissingen op verschillende niveaus (macro- en patiëntniveau) verstandig worden genomen. Als dat nodig is, kan het zorgsysteem worden aangepast, en kan er beter worden gestuurd op afspraken over kosten. Dit kan zowel binnen als buiten de militaire gezondheidszorg.

11. **Geef voor de volgende 6 uitspraken aan hoe belangrijk u de aandacht voor het gestelde vindt binnen de MGZ?**
(Schaal: ZB-zeer belangrijk / B-belangrijk / RB-redelijk belangrijk / EB-enigszins belangrijk / O-onbelangrijk)

Nr.	Uitspraak	ZB	B	RB	EB	O
1.	Kosten afspraken tussen zorgverzekeraars en zorginstellingen worden gebaseerd op waardegedreven zorg					
2.	De IT-omgeving stimuleert de zorgkwaliteit, communicatie en innovatie					
3.	Beslissingen worden makkelijker gemaakt met een overzichtelijk inzicht in de gezondheidsuitkomsten					
4.	Patiënt (én partner) en zorgverlener beslissen samen (shared decision making)					
5.	Relevante data zijn vindbaar, toegankelijk, uitwisselbaar en herbruikbaar					
6.	Overzichtelijke dashboards zijn betrouwbaar, beschikbaar en van hoge kwaliteit					
7.	Zorg is gericht op de behoefte en medische noodzaak van de patiënt.					

DEEL 3 – AFRONDING

In het vorige deel van de vragenlijst hebben wij u inzicht willen geven in de diverse elementen van WGZ, geplaatst in een militaire gezondheidszorg omgeving.

12. Op een schaal van 1 (helemaal niet bekend) tot 5 (buitengewoon bekend), in hoeverre voelt u zich ***nu*** meer bekend met waardegedreven zorg?
    Schaal: 1-2-3-4-5

1 - Helemaal niet bekend	2 - enigszins bekend	3 - redelijk bekend	4 - zeer bekend	5 - buitengewoon bekend

13. Vindt u dat (delen van) het concept van waardegedreven zorg van waarde zou kunnen zijn om in te voeren binnen de Nederlandse militaire gezondheidszorg?
    - Ja
    - Nee

    a. Bij nee: waarom niet?
    Score: open vraag
    b. Bij ja: ga naar volgende vragen:
    - Waar in de militaire gezondheidszorg zou WGZ van toepassing kunnen zijn?
        - Reguliere zorg (in Nederland)
        - Operationele zorg (tijdens missies en oefeningen)
        - Beide
    - In hoeverre zou de invoer van (delen van) WGZ wenselijk zijn binnen de militaire gezondheidszorg?
      Schaal: 1-2-3-4-5 (1=helemaal niet / 5=helemaal wel)

1 - enigszins wenselijk	2 - redelijk wenselijk	3 - wenselijk	4 - erg wenselijk	5 - buitengewoon wenselijk

- In hoeverre zou de invoer van (delen van) WGZ toepasbaar zijn binnen de militaire gezondheidszorg?
  Schaal: 1-2-3-4-5 (1=helemaal niet / 5=helemaal wel)

1 - enigszins toepasbaar	2 - redelijk toepasbaar	3 - toepasbaar	4 - erg toepasbaar	5 - buitengewoon toepasbaar

- Zijn er elementen/aspecten uit de beschrijvingen en beantwoorde vragen die in het oog springen en voor u een hoge prioriteit zouden moeten krijgen?
  Score: open vraag

Wij hopen dat u zich in zekere mate vertrouwd bent gaan voelen met het concept van WGZ.

14. Stel dat (delen van) het waardegedreven zorgmodel geïmplementeerd zou worden in de Nederlandse militaire gezondheidszorg. Wie vindt u dat dit initiatief zou moeten nemen?
    - Bottom-up door het directe zorgteam (artsen, verpleegkundigen, etc.) en patiënten(vertegenwoordiging)
    - Top-down door het indirecte zorgteam (administratief personeel, managers, leiders, etc.)

- Beide zorgteams in samenwerking met elkaar

Als u een bijdrage zou willen leveren bij de implementatie van waardegedreven zorg binnen de Nederlandse militaire gezondheidszorg, bijvoorbeeld met ondersteuning bij projecten. Neem dan contact op met Kol Henk van der Wal via civmilcetc@mindef.nl
