# Supplementary material for: Desirability and feasibility of value-based healthcare in the Dutch Military Health System: a cross-sectional study
Source: BMC Health Serv Res. 2026 Apr 11;26:735. doi: 10.1186/s12913-026-14517-y (PMC13196245; doi:10.1186/s12913-026-14517-y)
Supplement: Supplementary file 2 — Supplementary Material 2 [file 12913_2026_14517_MOESM2_ESM.pdf]

Supplementary material 2 (S2) - Questionnaire with English translation

PART 1 – INTRODUCTION

Brief introduction on Value-Based Healthcare (VBHC) and Military Health System (MHS)

The concept of Value-Based Healthcare was introduced in 2006 by Prof Michael Porter and Prof Elisabeth Teisberg. In brief, VBHC is about providing patients with the best possible care – care that they themselves value – in an improved relationship to the actual costs incurred. VBHC is playing an increasingly important role in Dutch healthcare. Patients are more actively involved in their care process and are encouraged to discuss their specific health goals with their care professional. Research indicates to benefits for both patients and professionals, and it contributes to sustainability in health care.

In contrast, the Dutch MHS is primarily focused on generating and sustaining operational readiness by ensuring medical support capabilities for military personnel. This survey examines the feasibility of VBHC within the MHS, where the VBHC concept distinguishes itself, among other things, by making the patient an active member of the multidisciplinary care team and by focusing on personal care needs.

The multidisciplinary VBHC team includes the direct care team (e.g., doctors, nurses, dentists, physiotherapists, practice assistants), the indirect care team (e.g., administrative staff, managers, leaders, technical & logistics personnel), and the patient as care recipient. Patients are experts in their own lives and best placed to say what valuable care means to them. Depending on the medical condition, the patient's partner, parents, children and/or informal carers may also be involved. The separation between the direct and indirect care teams helps safeguard privacy and medical confidentiality. Patient involvement is actively encouraged.

We will start with four questions about your background in Defence and your familiarity with VBHC. Please tick the option that best applies.

1. Where are you employed within the Ministry of Defence?
    - I do not work within the MHS -> military patient (care recipient)
    - I work within the MHS as a member of a direct care team (care professional)
    - I work within the MHS as a member of indirect care team (care facilitator)

**If you are not working within the MHS, please choose 'military patient' in order to answer the questions from your role as a (potential) care recipient.*

2. How much total experience do you have in Defence?
    - 0 - 5 years
    - 5 - 15 years
    - 15+ years

3. What is the highest level of education you have completed?
    - No diploma
    - Primary education
    - VMBO, HAVO / VWO lower years, MBO1
    - HAVO, VWO, MBO2-4
    - Bachelor (HBO / WO)
    - Master (HBO / WO)
    - Doctorate (PhD or equivalent)

4. On a scale from 1 (not at all familiar) to 5 (extremely familiar), how familiar are you with VBHC?
   Scale: 1-2-3-4-5

**1 - Not at all familiar**	**2 – Slightly familiar**	**3 – Moderately familiar**	**4 - Very familiar**	**5 - Extremely familiar**

PART 2 - DESCRIPTIONS OF VALUE-BASED HEALTHCARE

Seven descriptions of WGZ in a military context

You will now see seven short descriptions of one or more aspects of VBHC as applied within the MHS. After each, please indicate how important you believe attention to the stated elements would be within the MHS.

In Question 1 you selected a role (care recipient, care professional, or care facilitator). Please answer the remainder of the questionnaire from the perspective of your selected role, based on your views and experience.

Description 1. The aim of military healthcare is to improve the readiness of the service member. This occurs within a military medical care pathway that includes care activities delivered in the Netherlands (regular care) and during deployments and exercises (operational care). Where possible, the patient and care professional decide together about diagnostics and treatment, based on medical necessity, the availability of resources and options, and the patient's needs.

To achieve the best health outcomes, both patient-reported outcomes (e.g., pain, mental well-being) and clinical data (e.g., blood pressure, temperature) are used by the care professional *and* the patient when making these choices. Achieving the best outcomes requires multidisciplinary care from a team that includes doctors and nurses, but also support personnel and commanders—and, not least, the patient and their partner.

5. **Please rate how important attention to each of the following would be within the MHS?**
   (Scale: VI-very important / I-important / MI-moderately important / SI-slightly important / NI-not important)

Nr.	Statement	VI	Ì	MI	SI	NI
1	Patients (and partner) and care professionals decide together (shared decision making).					
2	The multidisciplinary team shares responsibility for health outcomes.					
3	The patient is central.					
4	Patient-reported outcomes contribute to better health outcomes.					
5	The care pathway is known and visible in a dashboard.					

Description 2. Dashboards provide an at-a-glance view of medical activities along the military care pathway and of patient-reported outcomes, both in the Netherlands and during deployments and exercises. Dashboards help the patient and care professional align diagnostic and treatment choices with the patient's needs. They can also support quality improvement and the efficient use of (often scarce) healthcare resources. All of this is directed towards delivering the most appropriate care with the best possible health outcomes for the military patient. It is important that everyone in the military care pathway—especially the patient and partner—knows who the lead clinician is, who the point of contact is, and where 24/7 help is available.

6. **Please rate how important attention to each of the following would be within the MHS?** *(Scale: VI-very important / I-important / MI-moderately important / SI-slightly important / NI-not important)*

Nr.	Statement	VI	Ì	MI	SI	NI
1	Dashboards are also available to the patient.					
2	The multidisciplinary team collaborates with everyone in the patient's military care pathway.					
3	The military care pathway reflects the total package of care for the patient.					
4	It is always clear to the patient (and partner) who the lead clinician is.					
5	It is always clear to the patient (and partner) where 24/7 help can be obtained.					
6	Outcomes are displayed clearly and simply in dashboards.					

Description 3. Care professionals in military healthcare strive every day to improve the quality of care and to develop themselves. They consider it important to keep learning—not only from patients, but also from one another and from (inter)national civil-military healthcare systems. The multidisciplinary team shares health outcomes with other care professionals, commanders and organisations within the care pathway. Sharing is used to optimise and/or innovate care and is always directed at better health outcomes and quality of life for the patient. The leader (or leadership team) is responsible for this improvement process and for optimising outcomes and dashboards.

7. **Please rate how important attention to each of the following would be within the MHS?** *(Scale: VI-very important / I-important / MI-moderately important / SI-slightly important / NI-not important)*

Nr.	Statement	VI	Ì	MI	SI	NI
1	Care professionals continue to develop in their profession.					
2	The multidisciplinary team uses health outcomes to learn and improve.					
3	Research findings are also available to the patient.					
4	Patient (and partner) experiences are part of the learning and innovation process.					

5	The multidisciplinary team is also responsible for the efficient use of resources.					

Description 4. Defence requires service members to use military healthcare, which is delivered by the military medical service or, after referral, by civilian healthcare. The best care is delivered when the multidisciplinary team jointly takes responsibility for the entire military care pathway. Collaboration, trust, clarity and learning from mistakes are essential. Leadership shows courage and ambition to improve care and fosters a culture of continuous learning and improvement.

8. **Please rate how important attention to each of the following would be within the MHS?** *(Scale: VI-very important / I-important / MI-moderately important / SI-slightly important / NI-not important)*

Nr.	Statement	VI	Ì	MI	SI	NI
1	Trust, learning and innovation are important within the care team.					
2	Research is directed at better care for a specific patient group.					
3	Care delivery is a team performance.					
4	Research findings are available to other organisations.					
5	The leader is responsible for the climate within the care team.					

Description 5. The care team is responsible for a positive culture around the military care pathway. The leader must ensure that everyone shares responsibility for both health outcomes and costs. By achieving better outcomes, more efficient care and lower costs, additional care may become available. The team should also engage military leaders and policy-makers outside the military care structure in a culture focused on delivering the best care. These stakeholders can provide facilities—including financial support—to improve care and the health outcomes chosen by the service member (and partner).

9. **Please rate how important attention to each of the following would be within the MHS?** *(Scale: VI-very important / I-important / MI-moderately important / SI-slightly important / NI-not important)*

Nr.	Statement	VI	Ì	MI	SI	NI
1	Patients (and partner) are also members of the multidisciplinary team.					
2	The leader shows courage, ambition and takes responsibility.					
3	Military leaders and policy-makers are actively involved in the care process.					
4	The multidisciplinary team shares responsibility for costs.					

Description 6. Leadership and culture are important elements of team performance. Desired results can be viewed from two perspectives: the organisation and the military patient. Organisationally, the team works as one to achieve the best results—e.g., rapidly evacuating and treating the wounded with

the right care along the military care pathway. From the patient's perspective, we ask whether the outcomes are truly good, using patient-reported outcomes such as pain scales or work functioning. This approach applies when comparing with other (civilian) providers as well as when collaborating with international military partners during operations. Such an approach supports a culture of safety, learning and improvement.

10. Please rate how important attention to each of the following would be within the MHS? *(Scale: VI-very important / I-important / MI-moderately important / SI-slightly important / NI-not important)*

Nr.	Statement	VI	Ì	MI	SI	NI
1	Collaboration between the care team and network partners is important for exchanging knowledge and experience.					
2	Individual team members have insight into the entire care pathway.					
3	Chain and network partners help keep care patient-centred and streamlined.					
4	Patient-reported outcomes are important.					
5	Greater collaboration is needed with external and international (military) partners.					

Description 7. The military care pathway is supported by data so that care professionals, patients and others can make sound medical and organisational decisions. This is done securely through an IT system. An IT platform provides reliable information that is available in a timely way and visible in dashboards. In this manner, decisions can be made wisely at different levels (from system to individual patient). Where needed, the care system can be adjusted and agreements about costs can be better managed—both within and outside military healthcare.

11. Please rate how important attention to each of the following would be within the MHS? *(Scale: VI-very important / I-important / MI-moderately important / SI-slightly important / NI-not important)*

Nr.	Statement	VI	Ì	MI	SI	NI
1	Cost agreements between insurers and care organisations are based on VBHC.					
2	The IT environment supports care quality, communication and innovation.					
3	Decisions are easier when health outcomes are clearly displayed.					
4	Patient (and partner) and care professional decide together (shared decision making).					
5	Relevant data are findable, accessible, exchangeable and reusable.					
6	Clear dashboards are reliable, available and high quality.					
7	Care is driven by the patient's needs and medical necessity.					

PART 3 - CONCLUSION

In the previous section we introduced various elements of VBHC situated in military healthcare context.

12. On a scale from 1 (not at all familiar) to 5 (extremely familiar), how familiar are you with VBHC?
    Scale: 1-2-3-4-5

1 - Not at all familiar	2 – Slightly familiar	3 – Moderately familiar	4 - Very familiar	5 - Extremely familiar

13. Do you think that (parts of) the VBHC concept could be of value to implement within Dutch MHS?
    - Yes
    - No

a. If no: why not?
   Score: open question
b. If yes: answer the following questions:
   - Where in military healthcare might WGZ apply?
     - Regular care (in the Netherlands)
     - Operational care (during missions and exercises)
     - Both
   - How desirable would the introduction of (parts of) WGZ be within military healthcare?
     Score: 1-2-3-4-5 (1=not at all / 5=completely)

1 - Somewhat desirable	2 – Slightly desirable	3 - Desirable	4 - Very desirable	5 - Extremely desirable

   - To what extent would (parts of) WGZ be applicable within military healthcare?
     Score: 1-2-3-4-5 (1=not at all / 5=completely)

1 - Somewhat applicable	2 - Slightly applicable	3 - Applicable	4 - Very applicable	5 - Extremely applicable

   - Are there elements from the descriptions or items above that particularly stand out and should receive high priority?
     Score: open question

14. Suppose that (parts of) the VBHC model were to be implemented in the Dutch MHS. Who do you think should take the initiative?
    - Bottom-up: direct care team (e.g. doctors, nurses, etc.) and patients' representatives.
    - Top-down: indirect care team (e.g. administrative staff, managers, leaders, etc.).
    - Both care teams working together.

If you would like to help implement VBHC within Dutch MHS – for example by supporting projects – please contact Col Henk van der Wal at: civmilcetc@mindef.nl.
