# Supplementary material for: Desirability and feasibility of value-based healthcare in the Dutch Military Health System: a cross-sectional study
Source: BMC Health Serv Res. 2026 Apr 11;26:735. doi: 10.1186/s12913-026-14517-y (PMC13196245; doi:10.1186/s12913-026-14517-y)
Supplement: Supplementary file 3 — Supplementary Material 3 [file 12913_2026_14517_MOESM3_ESM.pdf]

Supplemental material 3 (S3) - Barriers to implementing VBHC in the Dutch MHS & thematic analysis summary

	(Paraphrased) explicit reason why VBHC would not be of value within the Dutch MHS
1	- Care should be independent of cost.<br>- Integration of health care providers should improve.
2	I feel like we are already doing this. In my opinion, the patient already has a lot of direction and is included in everything. You can read everything in your online file. And there is continuous improvement. If not, it could possibly be of value.
3	Because the concept stems from regulated competition and I believe that that has not benefited healthcare as a whole, in the broadest sense of the word. However, preventive care is less profitable than performed care and is therefore more interesting from a cost efficient point of view. The Dutch military healthcare should be geared to individual and operational deployed employees.
4	I am not interested in the patient's opinion, but in his functioning. I want measured function-specific improvements as an outcome measure.
5	Not useful enough for operational care purposes in the event of a major conflict. Principles are based on a civil environment.
6	There should be eye for the major differences between the operational care chain, which has different principles for each deployment, and the regular military care chain. Both have different goals and relate differently to each other and thus to these VBHC ideas. I am not against improvement and input from patients, but in the proper context of our military system and roles of professionals involved.
7	It is all still far too vague. The majority of patients and doctors are not waiting for yet another dashboard/app. In 10 years of Defense, I have seen 10 of them already and all 10 are no longer applied.
8	It is far too much viewed from the health angle, from ' management', technology and cost savings. As a soldier, I have absolutely no need for everyone to know what I have, especially in the military - besides, 'being sick' is detrimental to your career. Just make me better, I'm busy enough. There is also a discrepancy with the organization. You do talk about care within the military. As a military organization, you just want your men patched up and well and back in combat as soon as possible. In addition, I completely disagree that good care depends on a leader; leaders are also made by the team around him or her and no one is really waiting for just guts.
9	Care should not be value-based. All care is important and necessary.
10	A holistic approach to care is currently the best way to care for patients in peacetime. Given the shift to main task 1, the pursuit of such a holistic approach is no longer possible and will require a much more basic look at the care to be delivered. This will require a large amount of adaptability from military healthcare.
11	The military healthcare main goal in main task 1 is to provide battle power for the operational units. The interests of patient and operational battle power may intersect in these situations. If VBHC is to be introduced in Defense, a component for operational interests will have to be added to the considerations. In pure peace service, VBHC may be useful, but should be able to give way when main task 1 comes into the picture.
12	Wrong time. Health centers are on the verge of collapse with quantitative staff shortages or falling over (together with lack of leadership). Operationally the same. This should be the

	focus to fix, not flashy dashboards.
13	- This model is about money and not about the patient.<br>- Because of the integral care provided by Defense, it's possible that a decision is made where the patient is not the focus.

Thematic Analysis Summary

Theme	Frequency	Representative Quote
Mismatch with Military Context	5	Not useful enough for operational care purposes in the event of a major conflict.
Already Doing It / Redundant	1	I feel like we are already doing this... continuous improvement.
Resistance to Management Tools / Tech	3	Far too vague... I've seen 10 dashboards in 10 years, none survived.
Patient-Centeredness vs Functionality	2	I am not interested in the patient's opinion, but in his functioning.
Resource Constraints & Timing	2	Wrong time. Health centers are on the verge of collapse.
Philosophical/Moral Objections	3	This model is about money, not about the patient.
