# Supplementary material for: Desirability and feasibility of value-based healthcare in the Dutch Military Health System: a cross-sectional study
Source: BMC Health Serv Res. 2026 Apr 11;26:735. doi: 10.1186/s12913-026-14517-y (PMC13196245; doi:10.1186/s12913-026-14517-y)
Supplement: Supplementary file 4 — Supplementary Material 4 [file 12913_2026_14517_MOESM4_ESM.pdf]

Supplemental material 4 (S4) - Key priorities for implementing VBHC in the Dutch MHS & thematic analysis summary

	Elements that should be prioritized when implementing VBHC
1	- From regular care, more thought should be given to operational units. At sea, the situation is very different from that on land, and it regularly does not seem to come across that way.<br>- In addition, more consideration of the patient (also operationally) as to what is possible and whether it is wise for this person to travel, is necessary. So more multidisciplinary consultation on certain individuals is needed, even if the only medical person is a military nurse instead of a doctor. The nurse knows the person best at that moment. This can prevent possible repatriations.
2	- The entire care path must be enjoyed by the military within our own military healthcare (unless this expertise is not present), but no more tolerating/allowing or even encouraging leakage as now sometimes happens by (e.g. non military doctors) at some military medical services.<br>- In the statement, “The IT environment stimulates quality of care etc”, stimulates can often be replaced by “frustrates”. Major enablers: our own privacy lawyers and ICT.<br>- In the context of good leadership it is desirable to sometimes turn some departments to the “right”, not only to keep it workable but certainly also to be able to share data for training etc.
3	- In an information overload world, I think the unification of how dashboards can be found, read and edited is of great value.<br>- The wants/needs of my colleagues are often luxury interests and separate often from quality. The desire for 24/7 care for example costs a lot and seems unnecessary to me.
4	Start with regular care to engage patients from there to operational care forward.
5	Availability, reliability and deployability (JIT) of (medical) resources as elements of value addition.
6	Better data and better dashboards Get much more from synergy between already existing care elements
7	Offer better care and be active instead of reactive.
8	- More attention to SDM (*shared decision-making*) within all levels of the care team.<br>- More focus on working with the patient where the patient is a specialist of her/his own disease/health process.<br>- More openness to patients about different (civil) options for care and if/how it is reimbursed.
9	Communication.
10	- Dashboard would be easy to better streamline patient readmission, no loss of info if health care providers work in the same system.<br>- Cost perspective/policy is not something executive care providers should be concerned with, they should have clarity on what the referral options are and not what each treatment costs.
11	Dashboards and clear transfer between disciplines. This is often what is lacking.
12	That the patient is the focus and not operational availability. Often you see that someone needs to be recovered quickly because a mission, assignment or task is coming up. The patient may then be physically recovered, but the mental part may cause complaints.
13	That the patient and partner are more involved in the entire process.
14	The best care is provided when the multidisciplinary team takes joint responsibility for the

	entire military care pathway. Here, cooperation, trust, clarity and learning from mistakes are important.
15	Ensuring patient privacy. By collecting and sharing a lot of data (digitally), it is critical that this can be done in a protected and secure environment.
16	- Making the patient a part of the care process is nice, however there should be retention of the professional consultation.<br>- IT/work processes now partially support and adjustments can be made quickly provided the right path is taken. Does the healthcare provider/leader know all processes to innovate or influence. Privacy assurance is already a challenge in Holland because the patient has to give permission with every care provider, let alone internationally and the sensitivities with respect to “hacking”.<br>- Work atmosphere is created together, the leader has influence, but it is a team effort. Every generation has opportunities and challenges, in an implementation aspects can be included to support the implementation.
17	The patient must be the focus, not a healthcare provider.
18	- The value of shared decision making is overestimated in my view; often patients just want you as a doctor to tell them what to do (especially with simpler things). If you ask: ‘What do you think is going on?’, you often get the answer: ‘You are the doctor’.<br>- High priority should be the exchange of information between healthcare providers.<br>- Lower priority, as far as I'm concerned is, access to one's own files.
19	The care within defense is divided over many disks, difficult to bring them together within VBHC but certainly worth the challenge!
20	Clarity point of contact.
21	One of the questions is about leadership of a healthcare team. Each leader does that in his or her own way. Taking responsibility yes, but how the leadership proceeds is up to the leader.
22	First, one must get the entire genome chain in order within the operational military healthcare in order for the VBHC to really flourish within the whole military healthcare.
23	In particular, a distinction must be made between peacetime care and care in operational circumstances. In the latter case, it is very difficult in practice to document care properly, for example in emergencies, but VBHC plays an important role in managerial qualities. You often see that in the case of emergency care, the patient is not or hardly part of the care process but, on the contrary, plays a very large role when there is less urgency (in medical/social-medicine repatriation for example) since someone wants to finish the exercise/military mission. Also, the home front plays a major role and the care providers where the patient goes to in the Netherlands.
24	I think that the employer should at most serve as a guardian of the Gatekeeper Act and otherwise should not get any info about a patient/employee. And that deserves more attention because the employer also organizes the provision of care.
25	Education should be given to all care providers, including general military nurses who are on standby) on this issue.
26	Centralized hospital care (CMH) is not always patient (family) friendly due to distance.
27	Give the patient control over his care path and make resources available within the care network that the patient can use at the right time, in the right environment. If necessary, help the patient direct care. See also the RACT (*Resource Group Assertive Community Treatment*) model in mental health care.
28	Money should never be an obstacle to providing good care!

29	Good ICT support is necessary for good insight into relevant data for everyone.
30	The interest and safety of the patient should always prevail over the desire to make things more insightful, pragmatic and/or cheaper.
31	The most important thing in my eyes is patient care because of the high demands the Defense organization and country place on our military. Any form of cost savings/efficiency that VBHC could bring along should at all times be seen as a by-product and should never be the solitary reason for importation.
32	Involving Patients within the multidisciplinary team.
33	The dashboard and clarity of the care pathway for the patient.
34	The danger is that hard conclusions are drawn from soft outcome measures and low reliability using outcomes that only partially answer the question. This does not improve the final care but costs extra time, time that could be spent on healthcare in the first place!
35	Deciding together etc. is a lot harder in an operational situation. There is often no choice.
36	ICT.
37	ICT links.
38	I believe outcomes should be explained by the doctor rather than in coordination with the patient, they are the medical specialists.
39	I think a patient dashboard is important.
40	I get the impression that the context of operational health care is now seen from the perspective of main task 2. With deployment main task 1, I think other dimensions come into play that need to be considered. Even then, learning and improvement, leadership and culture are important, but decision-making is not focused on the individual patient but on “doing the most for the most”.
41	I wonder if the patient should always be able to participate in decision-making. In fact, I have case histories where the patient wants all kinds of things, but it is not possible within the military context. Deployable as a professional does not necessarily mean deployable as a professional in operational conditions. Patients don't always want to see this, but as a supervisor, this is precisely what is leading.
42	- In regular care, shared decision making has always been an important issue.<br>- In the operational setting, situations may apply where the group or organization has an interest. However, even here there are situations that can/should proceed for the individual according to VBHC
43	In an operational context VBHC does not fit.
44	If there is multi-disciplinary work with a lot of patient data, working according to privacy guidelines is a must. Information must be properly put away, secure and retrievable. Especially within defense where military personnel regularly change positions.
45	Inter(national), civilian and military health care institutions cooperation.
46	International cooperation with chain partners.
47	IT support.
48	Link with regular care is clear. Linkage operational healthcare is not feasible from my perspective. Then other norms and standards apply, especially under main task 1.

49	- Increase quality of care.<br>- More multidisciplinary cooperation.
50	Easy access to one's own medical file is highly desirable.
51	Military mental health care / psychological care.
52	Multidisciplinary approach to care.
53	Operational care is all about maintaining combat power. It may be difficult to apply VBHC in that. However, military personnel do physically demanding work and I strongly believe in lifelong (mentally) healthy living and exercise. In that, a link to the social network and long term are important. Within the regular military healthcare there can be more room for dialogue and cooperation within the team.
54	Overview by dashboard for the patient.
55	Patient decides on own health and the associated measures/means.
56	Patients should be more involved in their own treatment and transparent care path for the patient.
57	Patients must remain central, and not research/study.
58	Putting patients more central. More personalized care pathways, where physicians move away from mutual agreements in care institutions and protocols.
59	Collaboration of chains deciding together with patient multidisciplinary working with clear main practitioner as a kind of case manager
60	Role of commanders should not be too large, because of privacy ed. Especially when it comes to repatriation, care in Holland etc.
61	Shared decision making.
62	Shared decision making.
63	Value driven care and information provision. The combination enables patients to make informed choices.
64	VBHC is in my opinion not applicable in main task 1, perhaps in 2 and 3.
65	VBHC is of little added value in acute care on the battlefield (war). It can have a good place in regular care and aftercare. This is true for example in the Navy where they know the SMD (*this is the social services unit of the Dutch Navy*). Multidisciplinary in consultation with lgv (*the respondent probably used this abbreviation for medical or social service personnel at the SMD*) and pt (*patient*) with a great success rate. Start there.
66	We as a unit provide operational R1 care. I wonder if involving the patient in the care process is applicable in the acute phase.
67	Establish (mutual) trust by:<br>- Putting patients first .<br>- Communicating well and openly (not only during the treatment process, but also if we want to implement (parts) of VBHC).<br>- Availability of needed information for patient and care team (during the treatment process, but also so we can determine if and how we want to implement (parts of) VBHC).
68	Care pathway, referrals and results.

Thematic Analysis Summary

Theme	Frequency	Representative Quote
Patient-Centered Care & Shared Decision Making	20	Patients should be more involved in their own treatment and transparent care path for the patient.
Operational vs. Regular Care Context	15	VBHC is in my opinion not applicable in main task 1, perhaps in 2 and 3.
ICT, Dashboards & Data Integration	18	Good ICT support is necessary for good insight into relevant data for everyone.
Multidisciplinary Collaboration	10	The best care is provided when the multidisciplinary team takes joint responsibility for the entire military care pathway.
Privacy & Security	6	Ensuring patient privacy. By collecting and sharing a lot of data (digitally), it is critical that this can be done in a protected and secure environment.
Leadership, Culture & Education	8	Education should be given to all care providers, including general military nurses who are on standby) on this issue.
